# Spinal cord stimulation as a novel neuromodulation strategy for enhancing language recovery in post-stroke aphasia: a hypothesis

**DOI:** 10.3389/fnhum.2026.1911156

**Published:** 2026-08-19

**Authors:** Di Lu, Mingming Zhao, Xin Yan, Shuyun Wang, Zhitao Li, Feng Yin

**Affiliations:** 1 Department of Neurosurgery, Aerospace Center Hospital, Beijing, China; 2 Department of Neurosurgery, Peking University Aerospace School of Clinical Medicine, Beijing, China; 3 Department of Neurosurgery, Huanhu Hospital Affiliated to Tianjin Medical University, Tianjin, China

**Keywords:** aphasia, eSCS, neuromodulation, neuroplasticity, spinal cord stimulation, stroke, tsDCS, tsPCS

## Abstract

Post-stroke aphasia is a debilitating language disorder that significantly impairs communication and quality of life. Although speech therapy and non-invasive brain stimulation techniques such as repetitive transcranial magnetic stimulation (rTMS) and transcranial direct current stimulation (tDCS) have shown clinical benefit, treatment responses remain variable and incomplete. We hypothesize that spinal cord stimulation (SCS), encompassing invasive epidural SCS (eSCS) and two mechanistically distinct non-invasive transcutaneous modalities, transcutaneous spinal direct current stimulation (tsDCS) and transcutaneous spinal pulsed current stimulation (tsPCS), may enhance language recovery in patients with post-stroke aphasia. Unlike cortical stimulation, spinal neuromodulation may engage ascending sensory pathways and propriospinal networks that influence distributed cortical systems. A series of small-sample studies conducted by the same research team has suggested that tsDCS combined with language therapy may confer benefits in post-stroke aphasia. We propose that SCS may enhance cortical excitability and functional connectivity within residual language networks through spinal–cortical interactions, thereby augmenting neuroplasticity and improving language outcomes when paired with behavioral therapy. This hypothesis predicts measurable improvements in naming, fluency, and comprehension beyond standard rehabilitation. If confirmed, spinal neuromodulation could represent a preliminary but potentially important systems-level strategy for aphasia recovery. Future studies should evaluate mechanistic pathways, optimal stimulation parameters, and clinical efficacy in randomized trials.

## Introduction

Aphasia affects more than 40% of stroke survivors and is associated with persistent disability and reduced quality of life (Thye and Mirman, 2018). Post-stroke aphasia results predominantly from ischemic or hemorrhagic injury to the left-hemisphere language network, encompassing classical regions such as Broca’s area, Wernicke’s area, and the angular gyrus, as well as interconnected subcortical structures and white matter pathways (Leyton et al., 2016). Conventional speech-language therapy (SLT), including impairment-based approaches and functional communication approaches, remains the cornerstone of aphasia rehabilitation but frequently yields incomplete recovery, particularly in the chronic phase (Vitti and Hillis, 2021).

Current cortical stimulation approaches, including transcranial direct current stimulation (tDCS) and repetitive transcranial magnetic stimulation (rTMS), directly modulate excitability at localized cortical loci, primarily perilesional or contralesional language areas. Despite moderate clinical evidence, their effects on language outcomes remain variable and often modest, in part because aphasia recovery depends on distributed network reorganization that extends beyond any single cortical target (Hamilton et al., 2011). Spinal cord stimulation (SCS) offers a theoretically distinct mechanism: by modulating ascending sensory input at the spinal level, it may engage broader network dynamics—including premotor, sensorimotor, and associative cortical regions—through bottom-up thalamocortical projections, rather than directly targeting language cortex. Functional MRI, TMS, and EEG studies have collectively demonstrated that spinal cord stimulation can modulate cortical activity, excitability, and functional connectivity through bottom-up pathways (Moens et al., 2012; Schlaier et al., 2007; Goudman et al., 2020). This bottom-up engagement could, in principle, prime the distributed neural substrate for language relearning in a way that cortical stimulation alone cannot, particularly in patients whose perilesional cortex is extensively damaged and where network-level compensation is the primary recovery mechanism. The following hypothesis formalizes this reasoning.

SCS, historically developed for chronic pain management (Tronnier et al., 2011), has evolved into a neuromodulatory technique for which emerging evidence suggests it may influence supraspinal circuits beyond its established role in pain modulation. Epidural SCS (eSCS) is an invasive technique in which electrodes are permanently implanted in the epidural space under general anaesthesia, offering sustained, precisely controllable, and focal modulation of spinal circuitry. Transcutaneous spinal cord stimulation (tSCS) encompasses two non-invasive approaches that differ fundamentally in current modality and neural target. Transcutaneous spinal direct current stimulation (tsDCS) applies a weak constant direct current (1.5–2.5 mA) through surface electrodes placed over the spine and modulates sensory pathway excitability through subthreshold membrane polarisation, with particular effects on ascending somatosensory pathways and thalamocortical projections. Transcutaneous spinal pulsed current stimulation (tsPCS) delivers pulsed or alternating current at carrier frequencies of 5–10 kHz and acts primarily by activating motor axons and large-diameter posterior root afferents to enhance rhythmic motor output; its mechanism of action and predominant neural targets therefore differ substantially from tsDCS (Rahman et al., 2022).

Within this framework, the available direct evidence for spinal neuromodulation in aphasia derives mainly from tsDCS studies (Marangolo et al., 2017; Pisano et al., 2021). We propose that these tsDCS findings provide a meaningful basis for extending the hypothesis to eSCS, on the following rationale: tsDCS, being non-invasive and reliant on low-amplitude direct current to modulate sensory pathway excitability through polarisation, represents a lower-intensity and less spatially precise form of spinal sensory modulation. If even this comparatively blunt instrument can produce measurable language improvements through somatosensory pathway engagement, it is scientifically reasonable to hypothesize that eSCS—which delivers stimulation directly to the epidural space with greater spatial focality, higher current controllability, and the capacity for sustained chronic activation—would produce equal or stronger modulation of the same ascending spinal pathways. This reasoning follows a well-established translational logic in neuromodulation: non-invasive techniques serve as proof-of-concept for the underlying mechanism, while invasive approaches are subsequently investigated to optimize the therapeutic effect. Accordingly, we treat the tsDCS aphasia evidence as initial mechanistic support for the broader hypothesis that epidural SCS may enhance language recovery through sensory pathway modulation, while acknowledging that direct evidence for eSCS in aphasia does not yet exist and must be established in future trials.

Recent high-impact clinical evidence demonstrates that cervical epidural stimulation improves voluntary upper-limb motor function in chronic stroke survivors, even years after injury, providing evidence that spinal-level neuromodulation can engage residual corticospinal and cortical networks to drive functional recovery in the motor domain (Powell et al., 2023). Although motor and language networks are organized differently, the motor rehabilitation evidence nonetheless establishes a relevant background for spinal modulation of supraspinal circuits, and thus may lend indirect support for language-specific claims.

Importantly, during clinical application of eSCS for post-stroke central pain in our center, we observed that several patients with concomitant aphasia reported subjective improvements in speech fluency and word retrieval following activation of pulse generator. These convergent, unplanned clinical observations, together with the previous tsDCS studies by Marangolo and colleagues, prompted us to explore whether SCS may facilitate language recovery in post-stroke aphasia.

## The hypothesis

We hypothesize that SCS (especially eSCS) can enhance language recovery in post-stroke aphasia through bottom-up neuromodulation of ascending spinal pathways, which in turn facilitates cortical network reorganization and neuroplasticity in distributed language networks. This hypothesis diverges from current neuromodulation strategies in aphasia—such as tDCS and TMS—which primarily target discrete cortical regions thought to directly support language processing. Instead, we propose that SCS may modulate more extensive sensorimotor and associative networks that indirectly support language relearning, thereby providing a novel mechanistic route for enhancing rehabilitation outcomes.

The hypothesis builds upon two converging lines of evidence:

(1) SCS as a neuromodulation technique capable of altering spinal and supraspinal circuit excitability in motor and sensory rehabilitation applications, as evidenced by improvements in motor and sensorimotor function in peripheral and central nervous system disorders following SCS or transcutaneous spinal stimulation (Iversen et al., 2024). The eSCS and tsPCS have been shown to excite dorsal root afferents and modulate descending and ascending pathways that can prime supraspinal input and enhance volitional motor control after neurological injury; tsDCS, through direct current polarisation, modulates sensory pathway excitability and has been shown to influence ascending somatosensory and thalamocortical projections. Animal studies have shown that SCS activates supraspinal structures beyond its primary spinal-level targets: for example, 4 Hz SCS in a neuropathic pain model increased c-fos-positive cells in the nucleus raphe magnus (NRM), a node within the descending pain-inhibitory pathway, indicating engagement of brainstem pain-control circuitry (Maeda et al., 2009). In a pilot resting-state fMRI study of patients with post-stroke or traumatic brain injury-related spasticity, cervical eSCS was associated with altered functional connectivity within the sensorimotor network and between brainstem and cortical motor regions (Mayorova et al., 2023). Taken together, these findings indicate that SCS engages supraspinal neural circuits that extend beyond the spinal gate, including descending pain-modulatory pathways and motor networks relevant to neurological rehabilitation.
(2) Preliminary evidence in aphasia research, where tsDCS applied over thoracic spinal segments combined with language therapy showed enhanced verb retrieval in chronic aphasia patients compared with sham stimulation, suggesting an interaction between spinal input and language-related cortical activity (Marangolo et al., 2017). This early proof-of-concept signal, while encouraging, derives from a small pilot study (*n* = 14) and requires independent replication. In another small study (*n* = 10), patients with aphasia received tsDCS treatment, and their symptoms of apraxia of speech improved, with the effect generalizing to untrained language tasks (Pisano et al., 2021).

This hypothesis addresses a pressing clinical gap: current therapies (chiefly behavioral SLT, even when augmented with cortical stimulation adjuncts) often yield limited and plateauing improvements, particularly in chronic aphasia, leaving many patients with substantial residual communication deficits. Novel interventions are urgently needed to expand therapeutic efficacy. In addition, compared with non-invasive tsDCS, eSCS may provide more direct, sustained, and focal modulation of spinal circuitry, potentially translating into more robust improvements in language function.

The scientific logic of our hypothesis rests on the following causal assumptions:

- Ascending sensory facilitation mechanism

SCS increases afferent input through dorsal column pathways at cervical or thoracic levels, with direct electrophysiological evidence in humans confirming that SCS recruits post-synaptic dorsal column (PSDC) fibres ascending toward thalamic relay stations (Gmel et al., 2023). This augmented sensory volley is relayed via thalamocortical projections to cortical regions that interface with language networks. Specifically, cervical dorsal column input from the cuneate nucleus projects predominantly to the contralateral ventral posterolateral (VPL) nucleus of the thalamus (Boivie, 1978; Rustioni et al., 1979), from which somatosensory information is relayed to primary somatosensory cortex, premotor cortex, and supplementary motor area (SMA). The proposed engagement of language-associated regions—including prefrontal, temporal, and posterior parietal cortices—is hypothesized to proceed via corticothalamic feedback circuits involving the mediodorsal (MD) and pulvinar nuclei: once VPL-recipient somatosensory cortex is activated, reciprocal corticothalamic projections from sensorimotor cortex may recruit the MD and pulvinar nuclei, which in turn project broadly to frontal, temporal and parietal regions that interface with the distributed language network (Cappe et al., 2009). The pulvinar in particular has established projections to temporal (including the Wernicke’s area), parietal cortex (Maeshima and Osawa, 2018), and the MD nucleus projects to prefrontal cortex (Cappe et al., 2009), and both nuclei have been implicated in the modulation of cortical language areas. This corticothalamic re-entry mechanism is proposed as the most anatomically plausible route by which SCS-driven somatosensory activation could propagate to language network nodes, though it has not been directly tested in the context of spinal stimulation. An alternative and potentially complementary route exists through the spinothalamic tract (STT): SCS applied at the dorsal surface of the spinal cord may also modulate anterolateral pathway activity, and the STT is known to project directly to the intralaminar thalamic nuclei and the MD nucleus in addition to its primary termination in VPL (Krames and Foreman, 2007; Willis, 1985; Applebaum et al., 1979). This parallel STT-mediated route would provide a second anatomical channel through which SCS could engage associative thalamic nuclei connected to prefrontal and language-related cortices, and may act synergistically with the dorsal column pathway described above. While the precise anatomical mapping from dorsal column input through thalamic relay nuclei to canonical language regions including Broca’s area, Wernicke’s area, and the arcuate fasciculus remains incompletely characterized, this is acknowledged as a foundational gap and represents a primary target for future mechanistic investigation. It is important to note that the thalamocortical pathway description above constitutes a hypothesis about the most plausible anatomical route, not an empirically established causal chain. Additional study has shown that stimulation of the cervical spinal cord can induce supraspinal changes in cortical activation and network excitability (Gelenitis et al., 2025).

- Network integration mechanism

Post-stroke aphasia recovery increasingly is understood to depend on a distributed network of cortical and subcortical regions rather than isolated language gyri (Heiss and Thiel, 2006). Bilateral and perilesional networks involving premotor cortex, SMA and sensorimotor regions have been implicated in compensating for damaged core language regions, with longitudinal neuroimaging demonstrating that SMA and dorsal anterior cingulate cortex provide facilitatory input to the language network during recovery (Jiang et al., 2025). SCS may facilitate functional connectivity across these distributed circuits by modulating ascending sensory input that converges with cortical motor-speech networks, thus supporting network integration and compensatory reorganization. It should be noted that the specific language network components predicted to be most influenced by SCS — and whether dorsal or ventral language pathways would be preferentially affected—remain to be determined empirically.

- Priming for Hebbian plasticity

Rehabilitation-driven plasticity is dependent on synaptic mechanisms in which simultaneous activation strengthens connections (Hebbian plasticity). By boosting baseline excitability and increasing responsiveness of cortical networks, SCS may act as a neuromodulatory primer, augmenting the effects of language therapy. This idea parallels findings in motor rehabilitation where electrical stimulation enhances activity-dependent plasticity when paired with task practice: SCS has been shown to promote activity-dependent plasticity and facilitate sensorimotor pathway restoration through a combination of Hebbian and non-Hebbian mechanisms, including synaptic potentiation, interneuronal reorganization, and altered afferent–efferent coupling (Samejima et al., 2022; Li and Xia, 2026). Research has demonstrated that SCS increases the excitability of spinal neurons, stimulates axonal regeneration, and promotes synaptic plasticity (Guo et al., 2024). Importantly, whether motor rehabilitation mechanisms of activity-dependent plasticity apply equivalently to language network relearning—given the differences in cortical organization and network architecture—remains a critical open question that motivates the testable predictions proposed below.

These three components are best understood not as independent mechanisms but as sequential steps within a unified pathway: SCS augments ascending afferent drive through two parallel anatomical routes—the dorsal column pathway (via VPL, with corticothalamic re-entry through MD and pulvinar nuclei) and the spinothalamic tract (via intralaminar and MD nuclei)—(Mechanism 1) → this convergent thalamocortical input modulates sensorimotor and associative networks including prefrontal and parietal language-interface regions (Mechanism 2) → the resulting increase in cortical network excitability lowers the threshold for activity-dependent synaptic strengthening during language therapy (Mechanism 3).

Unlike traditional cortical stimulation approaches (e.g., tDCS, TMS) that directly modulate excitability in localized gyri, SCS engages distributed bottom-up pathways, thereby potentially influencing whole-network dynamics. This could allow greater flexibility in facilitating recovery, especially in cases where cortical targets are extensively damaged or where network compensation is necessary.

**Figure 1:**
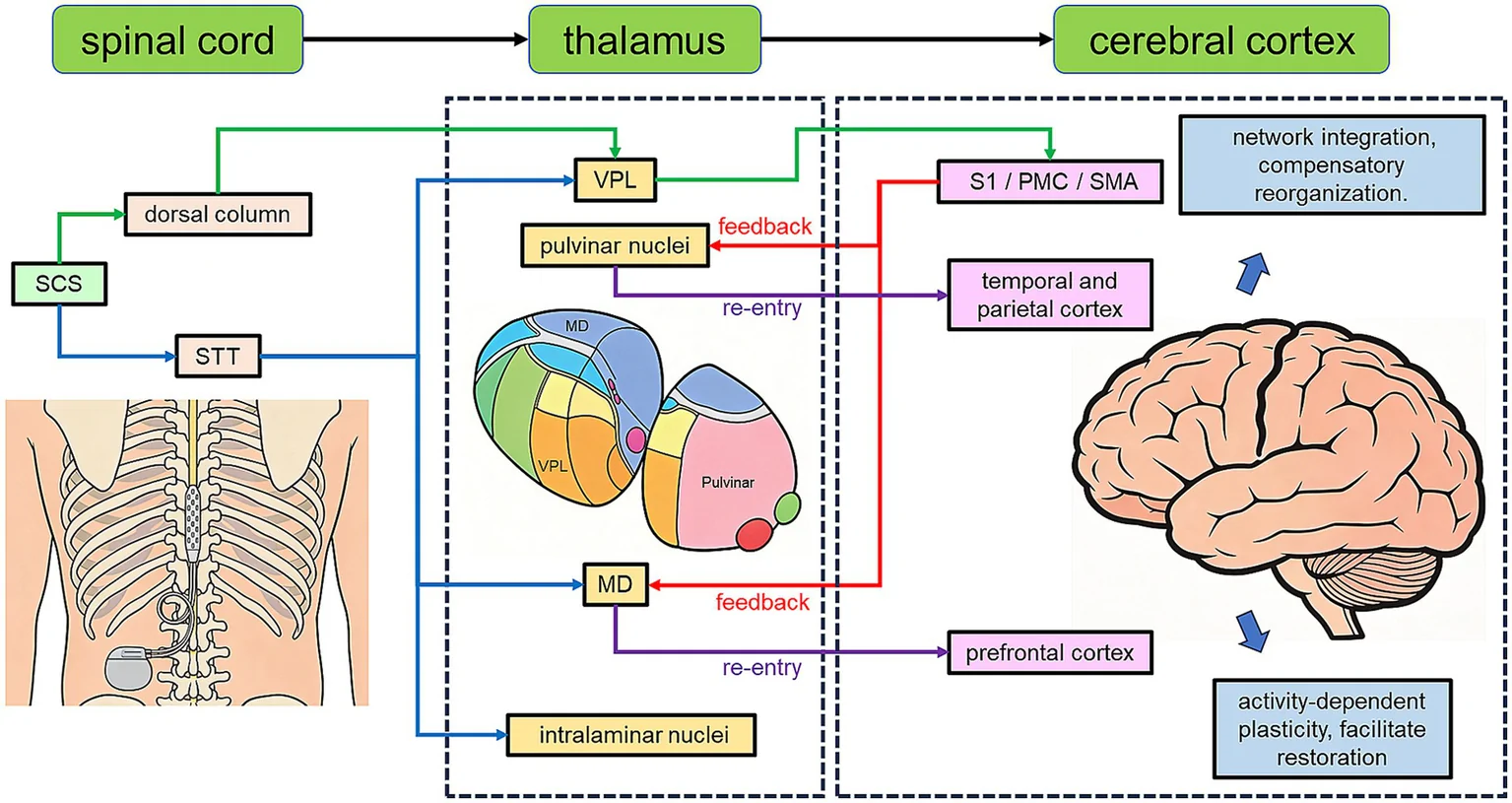
Schematic of the proposed sequential mechanism of SCS-enhanced language recovery in post-stroke aphasia.

In summary, the hypothesis posits a sequential framework (Figure 1):

SCS increases ascending afferent activity ➝ alters excitability in sensorimotor and associative language networks ➝ enhances distributed functional connectivity ➝ primes cortical plasticity ➝ improves response to language therapy.

This construct builds on existing neuromodulation frameworks while extending them into spinal-driven cortical facilitation, offering a new direction for aphasia rehabilitation research grounded in plausible neurophysiological mechanisms and early clinical observations.

## Evaluation of the hypothesis

The hypothesis that SCS could improve aphasia recovery, particularly in post-stroke patients, represents a novel extension of neuromodulation approaches traditionally focused on pain and motor rehabilitation. To evaluate its plausibility, we review the current supporting and conflicting evidence, and propose testable predictions for future empirical testing.

### Supporting evidence

#### Direct evidence—spinal neuromodulation in aphasia

The most direct evidence comes from three studies investigating tsDCS in chronic post-stroke aphasia, all from the same research group (Marangolo et al.). In a randomized, double-blind, sham-controlled crossover study (*n* = 14), anodal tsDCS applied over the thoracic spine during naming tasks produced greater improvement in verb naming than cathodal or sham conditions, with effects persisting one week after treatment; the authors proposed that tsDCS may influence ascending somatosensory pathways and thereby modulate sensorimotor cortical regions involved in language processing (Marangolo et al., 2017). A subsequent neuroimaging study from the same group (*n* = 16) extended this finding by demonstrating that tsDCS modulates a cerebellar-cortical network during verb recovery, suggesting that spinal stimulation engages distributed circuits beyond the primary somatosensory pathway (Marangolo et al., 2020). A further study (*n* = 10) showed that tsDCS was comparable or superior to cortical direct current stimulation in improving apraxia of speech, with gains generalizing to untrained language tasks (Pisano et al., 2021) (Table 1).

**Table 1:** Summary of available studies investigating spinal neuromodulation in aphasia.

Study	Design	Modality	Stimulation site	*n*	Principal findings	Key limitations
Marangolo et al. (2017)	Randomized, double-blind, sham-controlled crossover (3 conditions: anodal, cathodal, sham)	tsDCS	Thoracic spine (T10, spanning T9–T11)	14	Anodal tsDCS improved verb naming vs. cathodal and sham; effects persisted 1 week post-treatment; noun naming unaffected	Small sample; single research group; effects specific to verb naming; no neuroimaging
Marangolo et al. (2020)	Randomized, double-blind design with resting-state fMRI	tsDCS	Thoracic spine (T9–T10)	16	Anodal tsDCS enhanced connectivity in a cerebellar-cortical network; changes correlated with verb naming improvement; gains generalized to untreated verb tasks	Small sample; same research group; mechanistic interpretation of cerebellar-cortical pathway remains indirect; no independent replication
Pisano et al. (2021)	Randomized, double-blind, sham-controlled (anodal vs. sham)	tsDCS	Thoracic spine (T10, spanning T9–T11)	10	Anodal tsDCS improved articulatory accuracy (syllables, words, sentences); effects persisted at 1-week follow-up and generalized to untrained language tasks; comparable to tDCS in supplementary analysis	Small sample; single research group; supplementary tDCS comparison was not a randomized within-subject design; no long-term follow-up beyond 1 week

tsDCS, transcutaneous spinal direct current stimulation.

Indirect support within in this regard comes from a separate line of research: high cervical SCS has been reported to improve dysarthria in patients with Parkinson’s disease and multiple system atrophy, with improvements in voice quality and speech intelligibility observed across independent case series and small cohorts (Gong et al., 2022; Wang et al., 2022). While dysarthria and aphasia are distinct disorders, these findings suggest that SCS may exert a therapeutic effect on speech-related supraspinal circuits.

#### SCS-induced supraspinal cortical modulation

A mechanistically critical strand of evidence concerns whether SCS exerts supraspinal effects on cortical circuits at all, independent of any language-specific claim. Converging neuroimaging and neurophysiological data support this. Functional MRI evidence demonstrates that externalized SCS modulates activity in the thalamus and its connections to the anterior cingulate and cerebellum (Moens et al., 2012), consistent with the thalamocortical relay proposed in Mechanism 1. TMS-based studies show that SCS alters cortical excitability parameters—including motor-evoked potential amplitude and cortical silent period—in patients with chronic neuropathic pain (Schlaier et al., 2007), confirming that spinal stimulation produces measurable changes at the cortical level. EEG analyses further reveal that SCS induces changes in cortical power spectra and functional connectivity, with high-dose SCS preferentially amplifying the excitatory bottom-up pathway (Goudman et al., 2020). A comprehensive systematic review has synthesized this body of evidence, concluding that supraspinal mechanisms—including modulation of thalamic relay nuclei and engagement of anterior cingulate and somatosensory cortices—are plausible mediators of SCS effects on pain and cognition (Gartner et al., 2025). Taken together, these findings establish that the ascending pathway from spinal cord to cortex is accessible to SCS.

#### SCS in motor rehabilitation

Motor rehabilitation findings provide indirect but informative evidence that spinal neuromodulation can engage supraspinal networks, raising the possibility that similar mechanisms might contribute to language recovery. Cervical epidural stimulation has been shown to improve voluntary upper-limb motor function in chronic stroke survivors, even years after injury, providing proof-of-principle that spinal-level neuromodulation can engage residual corticospinal and cortical networks to drive functional recovery (Powell et al., 2023). More broadly, SCS has been shown to excite dorsal root afferents and modulate ascending and descending pathways that prime supraspinal input and enhance volitional motor control after neurological injury, with effects extending beyond pain modulation to influence broader neural circuits relevant to functional recovery (Iversen et al., 2024). While motor and language networks differ in their organisation, the motor evidence nonetheless establishes that spinal modulation can influence distributed cortical systems—a precondition that warrants investigation in the language domain.

### Evidence against or highlighting uncertainty

#### Limited direct evidence on SCS in aphasia

Despite promising pilot results, the volume and strength of direct evidence for SCS in aphasia remain very limited. The three tsDCS studies that provide the most direct evidence all originate from a single research group (Marangolo et al., 2017; Pisano et al., 2021; Marangolo et al., 2020), with sample sizes of 10–16 patients and no independent replication published to date. No group outside this team has, to our knowledge, investigated tsDCS or eSCS as a language intervention in aphasia. This absence of independent replication substantially constrains the interpretive weight of current findings, and generalizability, safety, and long-term efficacy of spinal neuromodulation in aphasia remain highly uncertain. The dysarthria case series noted in the Supporting Evidence section involve different patient populations and disorders, and cannot serve as direct evidence for aphasia rehabilitation (Gong et al., 2022; Wang et al., 2022).

In contrast, the broader neuromodulation literature shows that even for cortical stimulation (e.g., tDCS), evidence is mixed: systematic reviews have noted conflicting results across trials, with no clear consensus that tDCS substantially enhances aphasia recovery beyond naming accuracy and early rehabilitation effects (Elsner et al., 2020). This underscores that translation of neuromodulation benefits from motor or cognitive domains to language recovery is nontrivial.

#### Lack of mechanistic validation

A core challenge for the hypothesis is the current absence of direct mechanistic evidence causally linking spinal stimulation to altered neural dynamics within canonical language networks, such as Broca’s or Wernicke’s regions. While tsDCS may modulate somatosensory pathways that interact with sensorimotor cortices, there is no published evidence demonstrating that such modulation directly influences classical language networks or leads to robust reorganization of language processing areas.

Moreover, the clinical observations from our center that partially motivated this hypothesis, involving patients receiving SCS for post-stroke pain who reported speech improvements, are subject to alternative interpretations. Pain relief may reduce cognitive load, improve attention and concentration, or improve mood and reduce fatigue, each of which could independently improve language task performance without direct modulation of language networks. Additionally, generalized arousal effects or improved engagement with therapy following pain relief represent non-specific confounds. Future studies must incorporate appropriate control conditions—such as pain-matched control groups, double-blinding, sham stimulation, and standardized language assessments—to disentangle direct language network effects from indirect consequences of pain relief.

### Mechanistic reasonableness

The hypothesis is grounded in the broader neuroscientific notion that neuroplasticity across distributed networks can be enhanced by electrical stimulation. In the case of aphasia, recovery depends on the reorganization of language networks in both hemispheres following injury. If spinal stimulation can influence ascending pathways that in turn modulate cortical excitability or connectivity, it could theoretically contribute to a more favorable environment for language relearning.

However, this remains speculative, as direct measures (e.g., fMRI, electrophysiology) demonstrating SCS-induced changes in language network dynamics are currently lacking. Therefore, while the mechanism is plausible in principle, given known activity in sensorimotor pathways, it is not yet empirically validated for the proposed application.

### Testable predictions and research design

A scientifically rigorous hypothesis should generate specific, falsifiable predictions. The SCS hypothesis implies:

- Behavioral prediction: in patients with chronic post-stroke aphasia of mild-to-moderate severity (e.g., Western Aphasia Battery quotient 25–75 (Hula et al., 2010), a validated metric spanning fluent and non-fluent aphasia subtypes) and onset-to-enrollment interval of at least 6 months—to reduce the confound of spontaneous recovery—cervical eSCS or thoracic tsDCS combined with intensive naming therapy (minimum 10 sessions) will produce significantly greater improvements on pre-specified primary language outcomes—including standardized confrontation naming accuracy (e.g., Philadelphia Naming Test (Walker and Schwartz, 2012), Boston Naming Test), verb retrieval, and discourse-level measures (e.g., Cinderella story retelling)—compared to sham stimulation with equivalent behavioral therapy, at immediate post-treatment and at 4- and 12-week follow-up assessment points. This mild-to-moderate severity criterion applies to the initial proof-of-concept trial; patients with severe or complete aphasia are envisioned as candidates for later-phase studies or dual-indication designs (chronic refractory pain combined with aphasia), in which the surgical risk of eSCS is independently justified by a pain indication and language outcomes are evaluated as a secondary endpoint.
- Neurophysiological prediction: active SCS will produce measurable changes in cortical language network dynamics relative to sham, detectable by: (a) resting-state fMRI showing increased functional connectivity between perilesional language areas (e.g., left inferior frontal gyrus, left superior temporal sulcus) and sensorimotor/premotor regions, with a focus on frontotemporal and sensorimotor network nodes implicated in the thalamocortical pathway described above, given that such connectivity independently predicts therapy responsiveness (Falconer et al., 2024); (b) EEG showing increased gamma-band and theta-band synchronization during language tasks in left frontotemporal networks—assessable in real time and time-locked to stimulation onset—consistent with established oscillatory signatures of distributed language network integration (Doesburg et al., 2012); and (c) TMS-indexed cortical excitability changes (reduced resting motor threshold or altered silent period duration) in language-adjacent cortical regions, a paradigm with demonstrated sensitivity to language recovery interventions (Georgiou and Kambanaros, 2022).
- Dose–response prediction: language improvements will vary systematically with stimulation parameters, such that higher stimulation intensities (within safe limits), longer session durations, cervical versus thoracic electrode placement, and concurrent rather than sequential delivery with language therapy sessions will produce greater behavioral and neurophysiological effects. Although direct studies of eSCS for aphasia have not yet been reported, the cervical motor rehabilitation trial by Powell et al. provides a specific starting point: epidural leads targeted dorsal roots from C3 to T1, with participant-specific fixed pulse widths of 200 or 400 μs and contact-specific stimulation frequencies of 50–100 Hz (Powell et al., 2023). For tsDCS, parameters from the Marangolo et al. (2017) studies (thoracic placement, 2 mA, 20 min per session) offer a benchmark—systematic optimization of both modalities is needed to allow data-driven protocol refinement.
- Patient selection prediction: response magnitude will be moderated by baseline neurobiological characteristics: patients with preserved dorsal column and thalamocortical white matter integrity [assessed by diffusion tensor imaging of the arcuate fasciculus and related tracts (Moulton et al., 2019)], favorable lesion location, and higher baseline language network connectivity will show greater SCS-induced network changes and language gains than those with extensive subcortical damage disrupting the proposed ascending pathway.

Together, integrating clinical outcomes with neurophysiological and neuroimaging evidence would allow a comprehensive evaluation of whether spinal cord stimulation can meaningfully enhance language recovery and reshape neural networks involved in aphasia rehabilitation.

## Discussion

This study has proposed that spinal cord stimulation (SCS), particularly its capacity to modulate ascending sensory pathways, may offer a novel, bottom-up neuromodulatory strategy for enhancing language recovery in post-stroke aphasia. If empirically validated, this approach would carry preliminary but potentially important implications for the neuroscience of language recovery and the clinical practice of neurorehabilitation. Unlike conventional cortical stimulation that targets discrete language-related loci, the proposed strategy operates through spinal–cortical interactions that engage distributed network dynamics.

### A novel conceptual direction in aphasia rehabilitation

Traditionally, aphasia rehabilitation has focused on behavioral speech-language therapy and, more recently, cortical stimulation strategies (e.g., tDCS, TMS) to enhance plasticity in language-specific cortical areas (Fridriksson and Hillis, 2021; Cichon et al., 2021). The proposed hypothesis introduces a systems-level neuromodulatory direction in which modulation of ascending spinal afferents could influence distributed sensorimotor and associative networks that contribute to language recovery. Should SCS prove effective, it would expand the scope of rehabilitation targets from isolated cortical loci to integrated network dynamics, potentially offering alternative or complementary avenues for patients who have plateaued with standard therapies.

### Potential adjunctive role in chronic aphasia

A potential clinical application, if future evidence supports it, is that SCS could serve as an adjunctive intervention for individuals with chronic aphasia who demonstrate limited responsiveness to conventional speech-language therapy alone. While current aphasia treatments often yield modest improvements—particularly in chronic stages—SCS could, in principle, provide a neuromodulatory primer that enhances the efficacy of behavioral therapy, analogous to how stimulation paradigms in motor recovery have been used to augment rehabilitation outcomes. Rigorous trials are now needed to determine whether this mechanistic potential translates into clinical benefit for this population.

### SCS in the context of the broader neuromodulation landscape for aphasia

Positioning SCS appropriately requires acknowledging the breadth of neuromodulation strategies currently under investigation for aphasia rehabilitation. Beyond tDCS and rTMS, several other approaches are relevant. Vagus nerve stimulation (VNS) has demonstrated efficacy as an adjunct to upper-limb rehabilitation in chronic stroke—most recently confirmed in a randomized sham-controlled trial of a closed-loop VNS system (Hays et al., 2026)—and its application to post-stroke aphasia is now being tested in a registered clinical trial (NCT06403475) (ClinicalTrials.gov, 2024). Cerebellar stimulation is also emerging as a candidate approach: a recent randomized study demonstrated that right cerebellar tDCS paired with speech-language therapy improved quality of life across psychosocial, physical, and energy subdomains in chronic post-stroke aphasia, even though it did not significantly improve WAB-R language scores (Zheng et al., 2025); a completed randomized controlled trial of right cerebellar theta-burst stimulation found significant improvements in aphasia quotient, auditory comprehension, and repetition compared to sham, and attributed the effect to suppression of a right fronto-thalamic-cerebellar circuit (Dai et al., 2026); and an ongoing randomized, double-blind, sham-controlled trial (CeSAR; NCT05093673) is further evaluating cathodal cerebellar tDCS combined with semantic feature analysis therapy in chronic aphasia (Lammers et al., 2024). Multimodal neuromodulation strategies combining neural interfaces with behavioral therapy represent a further frontier. Against this landscape, SCS offers a distinct theoretical mechanism that differs from both cortical (tDCS, rTMS) and subcortical (VNS, cerebellar) approaches. From a practical standpoint, it is relevant that VNS in the cited aphasia trials also requires an implanted device—comparable in invasiveness to eSCS—whereas cerebellar and cortical approaches are largely non-invasive. The non-invasive tsDCS variant of spinal neuromodulation therefore occupies a lower-risk position comparable to cerebellar or cortical tDCS, while eSCS sits at the same invasiveness level as implanted VNS, a distinction that directly bears on patient selection and safety considerations. Whether SCS’s mechanistic distinctiveness translates into practical therapeutic advantages over these established approaches remains to be determined. Coupling SCS with cortical neuromodulation techniques could, in principle, synergistically enhance neural plasticity across multiple hierarchical levels of the nervous system (Cajigas and Vedantam, 2021).

### Broader applications to distributed network dysfunction

Beyond aphasia, if SCS were validated for language rehabilitation, this would suggest that spinal neuromodulation may be therapeutically relevant to a broader range of cognitive deficits arising from distributed network disruption. Evidence from spinal stimulation in other domains, such as cognition and attention modulation in chronic pain patients involving supraspinal regions including the anterior cingulate cortex and thalamus, points toward potential cognitive benefits mediated by spinal stimulation (Gartner et al., 2025). Extending this line of inquiry to other cognitive domains therefore represents a promising direction for future research.

### Safety, long-term effects, and patient selection

Safety considerations are especially important given that eSCS is an invasive intervention proposed here for a novel indication in a vulnerable clinical population. In its established indication of chronic pain, eSCS carries well-documented procedural and device-related risks, including infection (Falowski et al., 2019; Glinka Przybysz et al., 2025), lead migration (Dombovy-Johnson et al., 2022), hardware malfunction (Eldabe et al., 2016), and cerebrospinal fluid leak (Hussain et al., 2024). Post-stroke patients may face additional procedural risks arising from anticoagulation requirements, comorbid cardiovascular disease, and reduced physiological reserve. These risks must be systematically characterized in any future trial involving eSCS in aphasia patients.

A key ethical consideration for this population is patient selection and informed consent. For initial eSCS proof-of-concept trials, we recommend enrolling patients who already have a clinical indication for SCS (chronic refractory central pain) and concurrent partial aphasia. This dual-indication design justifies surgical risk on pain grounds, avoiding exposure to unproven language intervention alone; language outcomes are then a secondary endpoint. Patients with severe/complete aphasia should be included only after prospective capacity assessment by a clinician experienced in aphasia-related consent, using validated tools adapted for language impairment. Those unable to demonstrate adequate understanding of risks, procedures, or withdrawal rights—even with full communication supports—must be excluded, a criterion that aligns with standard ethical practice and should be explicitly stated in protocols. Because aphasia impairs comprehension of standard consent forms, all studies must include accessible consent procedures (e.g., simplified text, pictographs, supported decision-making) and involve speech-language pathologists. Since eSCS is approved only for chronic pain and is proposed for a novel indication, its risk–benefit profile is unknown and requires rigorous scrutiny. Non-invasive tsDCS has lower procedural risk and may be more suitable for initial proof-of-concept. Future work must systematically assess long-term durability of language gains, optimal parameters, and neurobiological differences between responders and non-responders.

### Implications for neuroscientific theory

On a theoretical level, confirmation of this hypothesis would stimulate a re-evaluation of how ascending sensory modulation contributes to higher-order cognitive processes. Current understanding of SCS mechanisms remains incomplete; although supraspinal effects have been proposed, such as modulation of somatosensory evoked responses and engagement of wider neural pathways, consensus is lacking (Goudman et al., 2021). Demonstrating that spinal stimulation can meaningfully influence language networks would expand foundational concepts in neuroplasticity, contributing to a more integrated view of bottom-up and top-down interactions in neural recovery.

In conclusion, this study advances a testable hypothesis that spinal neuromodulation may offer a bottom-up therapeutic direction for post-stroke aphasia. If empirically supported by independent replication and mechanistic investigation, spinal neuromodulation could introduce a novel bottom-up therapeutic direction for aphasia and contribute to a broader reconceptualization of how spinal and supraspinal circuits interact in cognitive and language recovery. Realizing this potential will require interdisciplinary work integrating neurophysiology, clinical neuroscience, and rehabilitation science, together with the rigorous trial methodology and safety evaluation that a new indication in a vulnerable population demands.

## Data Availability

The original contributions presented in the study are included in the article/supplementary material, further inquiries can be directed to the corresponding authors.
